# *MTHFR* Gene Mutations Correlate with White Matter Disease Burden and Predict Cerebrovascular Disease and Dementia

**DOI:** 10.3390/brainsci9090211

**Published:** 2019-08-22

**Authors:** Christian E. Cajavilca, Rajan R. Gadhia, Gustavo C. Román

**Affiliations:** 1 Vascular Neurology, Houston Methodist Hospital Neurological Institute, Houston, TX 77030, USA; 2 Alzheimer Clinic, Houston Methodist Hospital Neurological Institute, Houston, TX 77030, USA; 3 Weill Cornell Medical College, Department of Neurology, Cornell University, New York, NY 10065, USA

**Keywords:** *MTHFR* gene, dementia, homocysteine, Alzheimer’s disease

## Abstract

The incidence of dementia is on the rise and expected to continue to increase in the foreseeable future. Two of the most common subtypes of dementia are Alzheimer’s subtype and vascular dementia. Hyperhomocysteinemia has been shown to serve as a risk factor for dementia due to an associated blood–brain barrier dysfunction and subsequent small-vessel disease pathology. There are varying causes for hyperhomocysteinemia, including genetic and dietary, among others. We highlight the importance of identifying hyperhomocysteinemia as a potential etiologic and therapeutic target for the most common subtypes of dementia.

## 1. Introduction

Dementia is a neurological disorder that affects mainly the elderly population. According to the World Health Organization (WHO), the total number of new cases of dementia each year worldwide is nearly 10 million; there were an estimated 50 million people with dementia in 2017, and the total number of people with dementia is expected to increase to 74.7 million by 2030 [1,2]. It is expected that this number will continue to grow as life expectancy increases. Two of the most pertinent causes of dementia are Alzheimer’s disease (AD) and vascular dementia (VaD). Recent research suggests that cerebrovascular dysfunction is a major factor in both subtypes of dementia. In particular, blood–brain barrier (BBB) breakdown is an early biomarker of AD, independent of amyloid-β (Aβ) and tau protein [3,4]. A notable risk factor for developing dementia is hyperhomocysteinemia [5,6]. Elevated serum levels of total homocysteine (tHcy) are also associated with cardiovascular disease, including stroke. The most common genetic causes of hyperhomocysteinemia are mutations of the methylenetetrahydrofolate reductase (*MTHFR*) and cystathionine gamma-lyase (*CTH*) genes [7,8]. Other causes of elevated tHcy include advancing age, dietary B-vitamins deficiency, obstructive sleep apnea, smoking, *Helicobacter pylori* infection, and renal failure, among others [7,9,10,11].

## 2. MTHFR Genetic Variant

The *MTHFR* gene is located on chromosome 1 and serves an important role in the processing of amino acids. This gene produces an enzyme that catalyzes the conversion of 5,10-methylenetetrahydrofolate to 5-methyltetrahydrofolate, a co-substrate for homocysteine remethylation to methionine. There are two common polymorphic genetic variants of the *MTHFR* gene that can lead to impaired functioning of this enzyme resulting in hyperhomocysteinemia [12]; The most common of these genetic variants is the 677C>T polymorphism (C-to-T substitution at nucleotide 677) which encodes a thermolabile enzyme that is less active at high temperatures. These individuals tend to have higher homocysteine levels [12]. Another MTHFR variant is the 1298A>C. This variant, however, only results in hyperhomocysteinemia when it is found in combination with 677C>T.

Methionine is converted into *S*-adenosylmethionine (SAM), which is the major methyl donor within the cell. Methyl groups are important for DNA methylation, phosphatidylcholine synthesis, and protein synthesis. It has been reported that patients with late onset Alzheimer’s disease (LOAD) who are apolipoprotein E carriers have decreased levels of SAM in cerebrospinal fluid associated with MTHFR deficiency [7,13]. This hypomethylation induces up-regulation of AD-associated genes resulting in amyloid-B deposition and amyloid angiopathy, as well as tau pathology, in a mouse model [14]. DNA methylation is catalyzed by various types of DNA methyltransferases including DNMT1. Mutations in DNMT1 are pathologically associated with hypomethylation, which in turn results in a phenotype with learning, memory, and behavioral deficits [7].

## 3. Epidemiology

Approximately 25% of Hispanics and nearly 10–15% of North American Caucasians are estimated to be homozygous for the 677C>T polymorphism. This polymorphism is least common in individuals of African descent (6%) [12]. It is, however, common in other areas of the world, including northern China (20%), southern Italy (26%), and Mexico (32%) [13,15,16,17]. The MTHFR-A1298-C frequency in European Caucasians and African Americans is 31% and 15%, respectively [13]. 

## 4. Diagnosis

A simple peripheral venous blood draw can be used to measure total homocysteine levels. DNA is obtained from peripheral blood lymphocytes for analyses of MTHFR–C667T and MTHFR–A1298C homozygous and heterozygous polymorphisms. DNA analyses are completed using PCR amplification, followed by restriction analyses (diagnostic sensitivity >99% for both). Vitamin B12 levels and folate should be checked concurrently. Active vitamin B12 binds to transcobalamin, also referred to holotranscobalamin, which is the form available to cells [18]. Transcobalamin should also be measured, as total normal serum B12 levels can sometimes mask a true underlying deficiency [7]. Genetic testing of the *MTHFR* gene may be used to confirm a possible hereditary cause of hyperhomocysteinemia, secondary to MTHFR deficiency. The American College of Medical Genetics recommends against the use of genetic testing as part of routine evaluation of patients with thrombophilia. However, early detection of hyperhomocysteinemia in patients with cognitive decline and stroke risk factors may be beneficial as a treatable etiology to halt progression. Brain magnetic resonance imaging (MRI) can also be used to show white matter hyperintensities. Lesions in the periventricular area and frontal lobe areas have been found to be associated with elevated homocysteine levels in stroke patients [19]. Cerebrospinal fluid analyses of patients with Alzheimer’s disease who are Apo Ɛ4 carriers have also been shown to have low levels of SAM; however, this is not routine practice for work up and diagnosis [7].

## 5. Susceptibility to Dementia

Various studies have reported the association of dementia with elevated levels of homocysteine and concurrently low folate and vitamin B12 levels. Not surprisingly, other groups have found conflicting associations in conducted meta-analyses [20,21]. One such meta-analysis of 31 randomized placebo-controlled trials did not show convincing evidence of a cognitive benefit of lowering homocysteine [21]. Recent studies have found a folate-related locus in the methylenetetrahydrofolate dehydrogenase 1-like (*MTHFD1L*) gene located on chromosome 6, known as an Alzheimer’s disease linkage region. This gene is also involved in the homocysteine pathway and has been associated with late onset Alzheimer’s disease [13]. Adequate levels of folate and B12 are needed to maintain not only normal homocysteine levels, but proper methylation reaction. Deficient levels of these vitamins create a neurotoxic effect due to hyperhomocysteinemia, which in turn activates the *N*-methyl-d-aspartate receptor, ultimately leading to cell death. It has been postulated that ischemic insults to specific areas of the brain such as the hippocampus may trigger deposition of B-amyloid plaques and neurofibrillary tangles and eventually lead clinically to the onset of Alzheimer’s disease [22].

People affected by vascular dementia have also demonstrated an increase frequency of the C667T gene polymorphism as noted in a meta-analysis of 11 studies with strong association in the Asian population. This study also showed an association with coronary heart disease, ischemic stroke, hypertension, and diabetic nephropathy, all of which are risk factors for vascular dementia. The authors of this study considered that variations in this gene should be considered as a predisposing factor for development of this type of dementia [23].

Another reported consequence of elevated levels of homocysteine is the structural effect in the brain. It is well accepted that the orbitofrontal cortex is involved in cognitive function and emotional processing. In patients with hyperhomocysteinemia related to MTHFR mutations, there is a reported decrease in volume in the orbitofrontal cortex [24]. Reduced activity of MTHFR has also been noted to have an effect on the deregulation of the amyloid-β protein, which may provide another mechanism in the development of dementia [25]. Structural volume changes in the brain in relationship with homocysteine levels have been cited as well and may also contribute to dementia [26]. Other studies have reported temporal lobe atrophy in patients with Alzheimer’s disease when in association with hyperhomocysteinemia [27]. The vascular anatomy is also affected due to activation of inflammatory cells in an environment of elevated homocysteine [28]. Patients with Alzheimer disease often have evidence of small vessel injury in the white matter seen on imaging studies, which are thought to contribute to the clinical presentation of cognitive impairment and dementia [29]. Elevated levels of homocysteine are related to Aβ, which can also result in vascular toxicity [30]. Homocysteine levels of >14 μmol/L are associated with nearly 50% risk of developing Alzheimer’s disease [5].

Despite the relationship between hyperhomocysteinemia and dementia, studies have not shown a consistent association in all populations. The association of dementia and MTHFR polymorphism seems to be more predominant in certain populations, such as in Japan where the C667T genetic variant was associated with senile cognitive decline in men [13]. Individuals with MTHFR polymorphism in the American, Italian, Dutch, and Brazilian populations have found no significant susceptibility to dementia [13]. 

## 6. Small Vessel Disease and Stroke

As mentioned previously, high levels of homocysteine have been associated with Alzheimer’s disease and also with ischemic stroke. The China Stroke Primary Prevention (CSPPT) trial recruited patients with MTHFR mutations and found a significant reduction of the relative risk of first stroke to 21% in patients treated with folic acid and enalapril [31]. This trial suggested a relationship between small-vessel disease and stroke with MTHFR polymorphism in patients with underlying cognitive impairment and Alzheimer’s disease. There is also a suggestion that high levels of homocysteine may have a higher risk of presenting with worse clinical stroke symptoms defined as an NIHSS > 5 [32]. High serum levels of homocysteine appear to have a closer relationship to small vessel stroke than large artery or cardioembolic strokes, and consideration of treatment in this population should not be overlooked [33].

At the Houston Methodist Hospital, we studied a cohort of 48 female and 45 male patients (mean age 74.3 years) with diagnoses of mild cognitive impairment or dementia. DNA samples were obtained for MTHFR-C667T and -A1298C gene polymorphisms in all patients. All patients underwent brain magnetic resonance imaging (MRI), and we utilized the Fazekas grading scale [34,35,36] to analyze white matter changes. This scale has been validated histopathologically and provides a more objective grading system [37]. Within our cohort, 87.9% of patients with cognitive impairment had confluent white matter lesions (Fazekas grade 2 or higher). Of these patients, 91.3% had MTHFR mutations. These results indicate a statistically significant correlation between MTHFR polymorphism, dementia, and significant white matter disease burden etiologically presumed to be small-vessel disease, however, with a limitation of only a cognitively impaired population. A more comprehensive study population, including a control population without underlying cognitive impairment, is being planned for future study.

## 7. Management

Various randomized trials and meta-analysis have shown a significant reduction in the relative risk of stroke by 23% with the use of vitamin B12, folic acid, and pyridoxine [7]. These studies vary in dose, type and duration of supplementation which may contribute to the different outcomes. However, special consideration should be taken in patients with impaired renal function as cyanocobalamin may accelerate a functional decline [38]. Daily supplementation with 0.5 to 5 mg of folic acid and approximately 0.5 mg of methylcobalamin would be expected to reduce homocysteine levels found in typical Western populations on average by about one third [22]. There is a direct relationship of homocysteine levels and cognitive decline. Several studies [3,5,9,27,39,40,41] have showed a decline in memory when tHcy levels are higher than 11 µmol/L. In patients with adequate vitamin B12 but elevated homocysteine, administration of folic acid lowered homocysteine levels by 26%. Folic acid has been reported to improve information processing speed, sensorimotor speed, and complex speed [3]. As mentioned previously, DNA methyltransferases catalyzed DNA methylation. High levels of folate and vitamin B12 promote this methylation process [7]. Several countries have made mandatory the addition of folic acid fortification in certain foods, and it has been postulated that vitamin B12 should also be considered to reduce the incidence of dementia [7]. Lastly, clinicians should remember to address other factors that elevate homocysteine including obstructive sleep apnea, *Helicobacter pylori* infections, and renal disease. 

## 8. Conclusions

**Figure 1:**
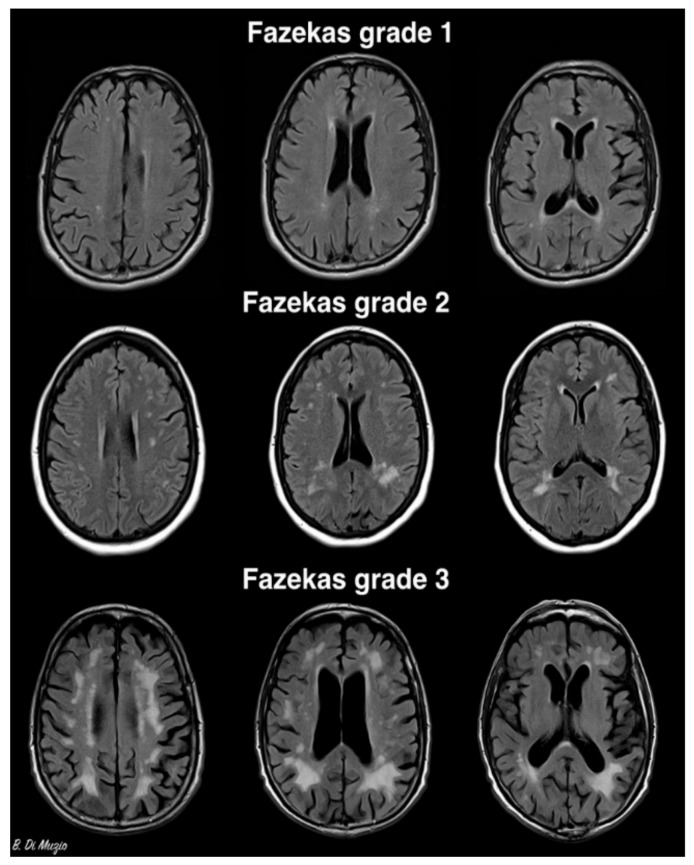
Grading scale based on size and confluence of white matter lesion. Grade 1: thin lining. Grade 2: small confluent areas in periventricular area. Grade 3: large confluent areas extending in deep white matter. Case courtesy of Dr Bruno Di Muzio, Radiopaedia.org, rID: 36927.

Hyperhomocysteinemia in elderly patients is a treatable risk factor for cerebrovascular disease and dementia. In the setting of MTHFR mutations it is often associated with cerebrovascular small-vessel disease presenting on imaging with higher ischemic white matter disease burden (Figure 1, Case courtesy of Dr Bruno Di Muzio, Radiopaedia.org, rID: 36927.), which usually responds to B-group vitamin treatment. Further studies are needed to determine causality between MTHFR mutations and subsequent neurological diagnoses, in particular, late-onset Alzheimer’s disease.
